# In Vitro Differentiation of Human Umbilical Cord Blood CD133^+^Cells into Insulin Producing Cells in Co-Culture with Rat Pancreatic Mesenchymal Stem Cells

**DOI:** 10.22074/cellj.2016.3717

**Published:** 2015-07-11

**Authors:** Fazel Sahraneshin Samani, Marzieh Ebrahimi, Tahereh Zandieh, Reyhaneh Khoshchehreh, Mohamadreza Baghaban Eslaminejad, Nasser Aghdami, Hossein Baharvand

**Affiliations:** 1 Department of Stem Cells and Developmental Biology at Cell Science Research Center, Royan Institute for Stem Cell Biology and Technology, ACECR, Tehran, Iran; 2 Department of Developmental Biology, University of Science and Culture, ACECR, Tehran, Iran; 3 Department of Regenerative Biomedicine at Cell Science Research Center, Royan Institute for Stem Cell Biology and Technology, ACECR, Tehran, Iran

**Keywords:** Mesenchymal Stem Cells, CD133^+^, Insulin Secreting Cells, Umbilical Cord

## Abstract

**Objective:**

Pancreatic stroma plays an important role in the induction of pancreatic cells by the use of close range signaling. In this respect, we presume that pancreatic mesenchymal cells (PMCs) as a fundamental factor of the stromal niche may have an effective role in differentiation of umbilical cord blood cluster of differentiation 133^+^ (UCB-CD133^+^) cells into newly-formed β-cells *in vitro*.

**Materials and Methods:**

This study is an experimental research. The UCB-CD133^+^cells were purified by magnetic activated cell sorting (MACS) and differentiated into insulin producing cells (IPCs) in co-culture, both directly and indirectly with rat PMCs. Immunocytochemistry and enzyme linked immune sorbent assay (ELISA) were used to determine expression and production of insulin and C-peptide at the protein level.

**Results:**

Our results demonstrated that UCB-CD133^+^differentiated into IPCs. Cells in islet-like clusters with (out) co-cultured with rat pancreatic stromal cells produced insulin and C-peptide and released them into the culture medium at the end of the induction protocol. However they did not respond well to glucose challenges.

**Conclusion:**

Rat PMCs possibly affect differentiation of UCB-CD133^+^cells into IPCs by increasing the number of immature β-cells.

## Introduction

Approximately 200 million people worldwide suffer from diabetes mellitus. This figure may double by 2025. The common treatment for type1 diabetes is daily injections of insulin (1). Transplantation of pancreatic islets is another method to treat diabetes, however worldwide shortage of transplant- ready islets, immune rejection and recurrent attacks against islets by underlying autoimmunity have yet to be resolved (2, 3). Application of insulin producing cells (IPCs) derived from adult stem cells is a potentially attractive strategy and may serve as a means to overcome many of the major issues that can complicate cell-based therapies, such as immune rejection and shortage of suitable donors (1). To this end, mesenchymal and hematopoietic stem cells from bone marrow and umbilical cord blood (UCB) represent valuable sources for differentiation into pancreatic β cells (4).

Many studies have reported that the microenvironment surrounding stem cells controls differentiation *in vivo* (5, 6). To our knowledge, stromal niches can be thought of as discrete anatomical sites that contain niche support cells which physically contact adjacent cells and influence stem cell behavior via close range signaling (6). It has been reported that pancreatic mesenchyme controls the timing of β-cell differentiation by secreted soluble factors. However, the identity of the soluble factors and mechanisms involved in regulating the development and function of the pancreas remains unknown (7). Other studies have reported that extract of rat pancreas could induce mesenchymal stem cell (MSC) differentiation into IPCs *in vitro* with concomitant increases of insulin. However the extract could not induce functionally mature pancreatic cells responsive to different concentrations of glucose (8-10).

Therefore, the purpose of our study was to investigate the differentiation of human UCB-cluster of differentiation 133^+^ (CD133^+^) cells into IPCs in co-culture with rat pancreatic MSCs (PMCs).

## Materials and Methods

### Isolation and culture of umbilical cord blood cluster of differentiation 133^+^ cells

This study is an experimental research. Fresh cord blood samples obtained from the Royan Public Cord Blood Bank were immediately diluted with HAES-Steril (Free flex, Germany) 10% at 1:5 (v/v) to accelerate red blood cell (RBC) sedimentation and facilitate isolation of cord blood mononuclear cells (MNCs). Subsequently, the MNCs were isolated using a ficoll density gradient (Inno-Train, Germany) and then washed twice in phosphate buffer saline (PBS, Invitrogen, USA) that contained 0.5% fetal bovine serum (FBS, Sigma, USA) and 2 mM ethylenediaminetetraacetic acid (EDTA, sigma, USA). Magnetic cell sorting (MACS, Milteny Biotech, Bergisch Gladbach, Germany) was used for isolation of CD133^+^ cells according to the manufacturer’s guidelines. Briefly, 100 μL of FcR blocking and 100 μL of CD133 microbeads were added to at least 1×10^8^ MNCs/300 μL, then mixed and incubated for 30 minutes at 2-8˚C. After washing with PBS that contained 0.5% FBS and 2 mM EDTA, cells were resuspended in 500 μL of the same PBS solution. A MACS column was used to isolate highly pure CD133^+^ cells from the cell suspension according to a data sheet. A sample fraction of the purified cells was checked for viability, cell number, morphology and purity.

### Isolation and culture of rat pancreatic mesenchymal stem cells

We isolated rat PMCs by removing the pancreases of 7-day postnatal Wistar rats (n=5) according to a protocol approved by the Institutional Review Board and Institutional Ethical Committee at Royan Institute. Briefly, pancreas tissue was diced into 1 mm³ pieces in RPMI 1640 that contained 1 mg/ml collagenase type 1a (Sigma, Germany) using sterile blades and incubated for 90 minutes at 37˚C. The collagenase solution was inactivated with RPMI 1640 supplemented with 15% FBS. Cell clumps and undissociated tissue were removed by passing the tissue through a nylon mesh ﬁlter (100 mm). Washed cells were resuspended in RPMI 1640 (Sigma, Germany) supplemented with 10% FBS, 100 IU/ml penicillin (Invitrogen, Germany), 100 mg/ml streptomycin (Invitrogen, USA) and 2 mM L-glutamine (Invitrogen, USA). Cells were then seeded in 25 cm^2^ culture flasks (Cellstar, Greiner, Germany). Two days later, the medium was changed to remove non-adherent cells. When cells reached proper confluency they were trypsinized (5 mg trypsin/ml PBS), washed, resuspended in 20 ml medium and cultured in 75 cm^2^ flasks.

### Flow cytometry analysis

Rat PMCs were harvested by treatment with 0.25% trypsin (Gibco, Germany), washed with PBS (pH=7.4) and labeled directly with anti-rat CD90-fluorescein isothiocyanate (FITC), CD44- FITC, CD45-phycoerythrin (PE), and CD11b-PE. After washing, PMCs were fixed with 4% paraformaldehyde (sigma, Germany) for 20 minutes. The specific fluorescence of 20000 cells was analyzed by FACSCalibur (Becton Dickinson, Temse, Belgium) using WinMDI 2.9 software.

### Osteogenic and adipogenic differentiation

Rat PMCs at passage three were used for osteogenic and adipogenic differentiation. Rat PMCs were cultured for 21 days in Dulbecco’s Modified Eagle Medium (DMEM) that contained 10% FBS, 50 mg/ml ascorbic acid 2-phosphate, 10 nM dexamethasone and 10 mM b-glycerol phosphate (all purchased from Sigma, Germany). Differentiation was confirmed by observation of extracellular matrix calcification using alizarin red staining.

DMEM-high glucose supplemented with 10% FBS, 60 mM indomethacin, 10 nM dexamethasone and 10 mg/ml acid ascorbic (all from Sigma, USA) was used as the differentiation medium for adipogenic differentiation. Passage-3 rat PMCs were used for these experiments. Differentiation media were changed every 3 days; after 21 days, cells were fixed with cold 10% formalin (sigma, Germany) for 1 hour, then washed with water and stained with oil-red solution (Sigma, Germany) for 2 hours at room temperature. The presence of intra-cellular lipid droplets in the cytoplasm was observed with an optical microscope.

### Differentiation of CD133^+^ cells into insulin producing cells

**Fig.1:**
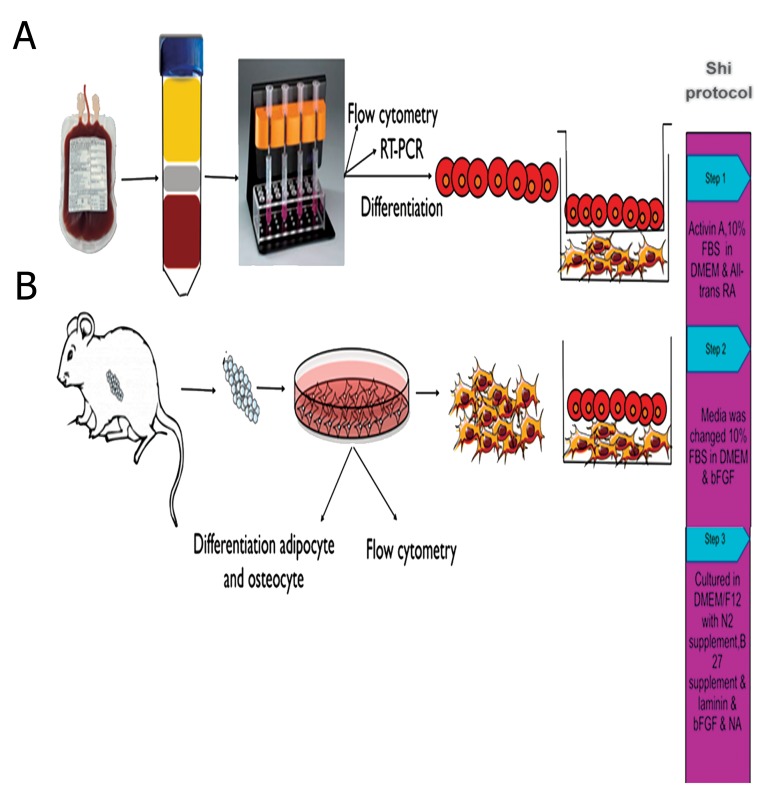
Schematic diagram of the experiment. Isolation and characterization of A. Umbilical cord blood cluster of differentiation 133^+^ , B. Rat pancreatic mesenchymal stem cells, C. Culture type, C1; Transwell culture, C2; Direct co-culture and D. Method of differentiation into insulin producing cells. FBS; Fetal bovine serum, DMEM; Dulbecco’s Modified Eagle’s medium, bFGF; Basic fibroblast growth factor, RA; Retinoic acid, NA; Nicotinamide and RT-PCR; Reverse transcription-polymerase chain reaction.

Figure 1 illustrates the protocol to induce β-cell differentiation in the presence or absence of PMCs. Briefly, 1×10^6^ CD133^+^ cells were plated into six-well plates in 10% FBS in DMEM according to the protocol by Shi et al. (11). Subsequently, 100 ng/ml activin A (Sigma, Germany) was added and cells were allowed to incubate at 37˚C, 5% CO_2_ and 95% humidity for 24 hours. At the end of the incubation time, the medium was changed to DMEM that included 10% FBS for 6-8 hours followed by addition of 1 μM all-trans retinoic acid (Sigma, Germany) for an extra 24 hours (step 1). The medium was changed to DMEM supplemented with 10% FBS and 10 ng/ml of basic fibroblast growth factor (bFGF, Sigma, Germany) for 3-5 days (step 2). Finally, cells were cultured in DMEM/F12 with N2 and B27 supplements (all from Gibco-BRL-USA), 1 μg/ml laminin (Sigma, Germany), 10 ng/ml bFGF and 10 mm nicotinamide (Sigma, Germany) for an additional 3-5 days (step 3).

### Co-culture of CD133^+^ cells with rat pancreatic mesenchymal stem cells

To investigate the effect of rat PMCs on differentiation of UCB-CD133^+^ into IPCs, expanded rat PMCs at passage-3 were plated in six-well plates and allowed to grow to approximately 80-90% confluency. Mitomycin C (Sigma-Aldrich, USA), at a concentration of 25 μg/ml, was subsequently added to the culture medium to prevent stromal cell proliferation and the cells were incubated at 37˚C, 5% CO_2_ and 95% humidity overnight. The next day, UCB-CD133^+^ cells were directly seeded on the inactivated stromal cell layer at a ratio of (1:1) or on the transwell system (Fig.1D). Then, the differentiation protocol was performed according to the Shi et al. (11) method.

### Immunocytochemistry

Differentiated cells were fixed with 4% paraformaldehyde for 20 minutes at 4˚C, washed several times and blocked for 30 minutes in 10% goat serum (Gibco, USA). Next, cells were allowed to incubate overnight with mouse anti-human insulin 1:500 (Sigma-Aldrich, USA) and mouse anti-human C-peptide 1:250 (Abcam, Cambridge, UK) as primary antibodies at 4˚C, followed 3 times washing with PBS. FITC-conjugated goat anti-mouse antibody (1:100, Sigma, Germany) was applied for 1 hour at 37˚C and subsequently washed by PBS to remove unwanted conjugated antibodies. Nuclei were counterstained with 4΄,6-diamidino- 2-phenylindole (DAPI) and cells were visualized using a fluorescence microscope (Olympus BX51, Japan).

### Enzyme-linked immunosorbent assay (ELISA)

In order to test whether the insulin released from differentiated cells was glucose-dependent, we used two glucose concentrations (2.5 mM and 27.5 mM) (12). The human insulin and C-peptide levels in culture supernatants were measured by a Human Insulin ELISA Kit and Human C-peptide ELISA Kit (Mercodia, Switzerland) according to the manufacturer’s instructions. The differentiated cells were pre-incubated in Krebs-Ringer buffer (120 mM NaCl, 5 mM KCl, 2.5 mM CaCl_2_, 1.1 mM MgCl_2_, 25 mM NaHCO_3_ and 0.1% bovine serum albumin) at 37˚C for 90 minutes. Then, Krebs-Ringer buffer (Sigma, Germany) was replaced by Krebs-Ringer buffer that contained 5 mM glucose at 37˚C for 15 minutes in order to determine the basal level of insulin and Cpeptide. To induce the release of insulin, the same differentiated cells were subsequently incubated with 27.5 mM glucose for an additional 15 minutes.

### Reverse transcription polymerase chain reaction (RT-PCR) analysis

The expressions of *CD133, OCT4* and *NANOG* were analyzed by RT-PCR. Briefly, total RNA was extracted from cells using RNX reagent (Sinagene, Iran) according to the manufacturer’s instructions. cDNA was generated by a cDNA Synthesis Kit (Fermentas, USA). cDNA was subjected to RTPCR using specific primers for *CD133, OCT4* and *NANOG* (Table 1). Glyceraldehyde 3-phosphate dehydrogenase (*GAPDH*) was used as an internal control. PCR conditions were: 94˚C for 4 minutes, 30 cycles of 94˚C for 1 minute, 60˚C for 45 seconds, 72˚C for 1 minute, and 72˚C for 10 minutes. PCR products were separated on a 2% agarose gel that contained ethidium bromide (Invitrogen, USA) and photographed.

### Real-time quantitative polymerase chain reaction (qRT-PCR)

The expressions of *GLUCAGON, INSULIN, PDX1* and *NKX6.1* were analyzed by qRT-PCR. Briefly, cells were harvested from differentiating plates and the total RNA was isolated from triplicate samples by an RNA Extraction Kit (TaKaRa, Japan). We used 100-500 ng for reverse transcription with a Prime Script II Strand cDNA Synthesis Kit (TaKaRa, Japan). PCR reactions were run in duplicate using 1/40^th^ of the cDNA per reaction and 400 nM forward and reverse primers with SYBR Green Master Mix (TaKaRa, Japan). RT-PCR was performed using the Rotor Gene 3000 (Corbett Research, Germany). QRT-PCRs were performed in duplicate for each sample primer set, and the mean of the three experiments was used as the relative quantification value. Relative gene expression was analyzed using the comparative Ct method, 2^-ΔΔCt^. All samples were normalized to the levels of *GAPDH*, which was used as the loading control. Primer sequences related to specific pancreatic genes are listed in table 1.

**Table 1:** Primers used for reverse transcription-polymerase chain reaction


Gene	5ˊ to 3ˊ	Annealing temperature	bp

GAPDH	F: CTC ATT TCC TGG TAT GAC AAC	58	224
	R: GA CTT CCT CTT GTG TTGCT		
GLUCAGON	F: CCA GAT CAT TCT CAG CTT CC	56	180
	R: GGC AAT GTT ATT CCT GTT CC		
INSULIN	F: AGC CTT TGT GAA CCA ACA CC	60	245
	R: GCT GGT AGA GGG AGC AGA TG		
PDX1	F: GGA TGA AGT CTA CCA AAG CTC AC	62	180
	R: CCA GAT CTT GAT GTG TCT CTC G		
NKX6.1	F: GTT CCT CCT CCT CCT CTT CCT C AAG	58	381
	R: ATC TGC TGT CCG GAA AAA G		
NANOG	F: AGC TAC AAA CAG GTG AAG AC	58	145
	R: GGT GGT AGG AAG AGT AAA GG		
OCT4	F: GTTCTATTTGGGAAGGTATTCAGC	60	323
	R: GTT ACA GAA CCA CAC TCG GA		
CD133	F: TAAGTACTATCGTCGAATGG	60	310
	R: TCAAGCAGTTTCAACATCAGC		


### Statistical analysis

The data are presented as mean ± standard error (SE). Each experiment was repeated in triplicate. Statistical significance was determined using the Univariate analysis of variance, two-way ANOVA and repeated measures test. We used the nonparametric Mann-Whitney test to compare the two differentiation groups (with and without co-culture). P values less than 0.05 were considered statistically significant.

## Results

### Human umbilical cord blood cluster of differentiation 133^+^ cells and rat mesenchymal stem cells isolation and characterization

**Fig.2:**
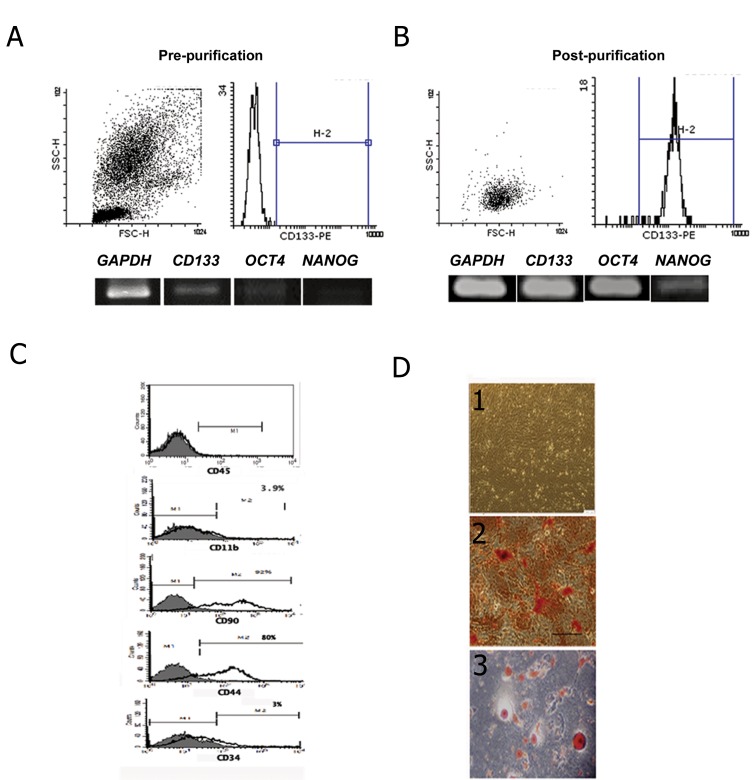
Isolation and characterization of umbilical cord blood cluster of differentiation 133^+^ and rat pancreatic mesenchymal stem cells. A, B. The percentage of UCB-CD133^+^ pre- and post- purification according to magnetic activated cell sorting technology (up), purified cells strongly expressed CD133, OCT4 and NANOG at the mRNA level according to real-time polymerase chain reaction (down), C. Flow cytometry analysis showing the expression of MSCs surface markers in rat pancreatic stromal cells at passage-3. Rat PMCs expressed CD44 and CD90. CD45, CD34 and CD11b were the negative markers. The percent positivity of each marker is indicated, D1. Morphology of rat PMCs at passage-3 by phase contrast microscopy, D2. Osteogenic differentiation of the test groups confirmed by Alizarin red staining and D3. Rat PMSCs differentiated into adipocytes which were confirmed by oil red staining (×200).

Figure 1 is a schematic diagram for isolation and differentiation of UCB-CD133^+^ and rat MSCs. CD133^+^ cells were isolated using MACS technology according to a Miltenyi Biotech protocol. The purified cells were passed twice through the MACS column to increase cell purity. The percentage of cells prior to purification was 0.1-0.4%, and reach to approximately 91%, after purification. Purified cells expressed strong bands of *CD133, OCT4* and *NANOG* compared to cord blood MNCs when detected by RT-PCR (Fig.2A, B).

Adherent cells derived from the pancreases of 7-day postnatal Wistar rats also showed typical fibroblast-like structure that rapidly reached confluence (7-10 days). These cells showed a similar phenotype and expression pattern to Bone Marrow-MSCs. They were positive for CD44 and CD90, negative for CD45 and CD11b (Fig.2C). The cells differentiated into osteocytes and adipocytes in differentiation medium (Fig.2D).

### Cell differentiation

Differentiation into IPCs was performed according to the Shi et al. (11) protocol (Fig.1D). The protocol included cell expansion and production of forgut endoderm cells by addition of activin A, followed by treatment with all-trans retinoic acid in the presence of serum, differentiation into posterior like cells in presence of bFGF and finally, differentiation into pancreas progenitor cells with laminin and nicotinamide.

**Fig.3:**
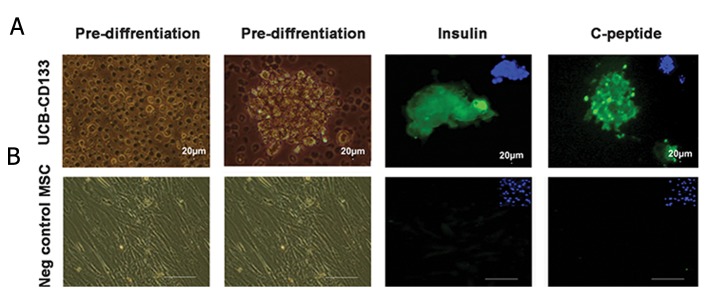
Immunofluorescence staining for insulin (FITC) and C-peptide (FITC) in differentiation umbilical cord blood cluster of differentiation 133^+^ (UCB-CD133^+^) cells. A. The bright field images of pre- and post-differentiation UCB-CD133^+^ cells. Expressions of insulin and C-peptide conjugate with FITC (green) and nucleus stained with DAPI and B. Bright field images of mesenchymal stem cells (MSCs) pre- and post-differentiation and lack of expressions of insulin and C-peptide in the cells (×100).

Throughout induction of cells with the Shi et al. (11) protocol, a number of changes appeared in cell morphology. The CD133^+^ cells expanded in the first step; in the second step, dense colonies formed which were positive for C-peptide and insulin in the final step (Fig.3A). Untreated CD133^+^ cells and PMCs were used as negative controls (Fig.3B).

### Effect of rat pancreatic mesenchymal stem cells on differentiation into insulin producing cells

In order to determine whether PMCs, as an effective factor in the pancreas niche, could increase efficiency of IPCs production, we directly and indirectly co-cultured the cells with rat PMCs (Fig.1C).

**Fig.4:**
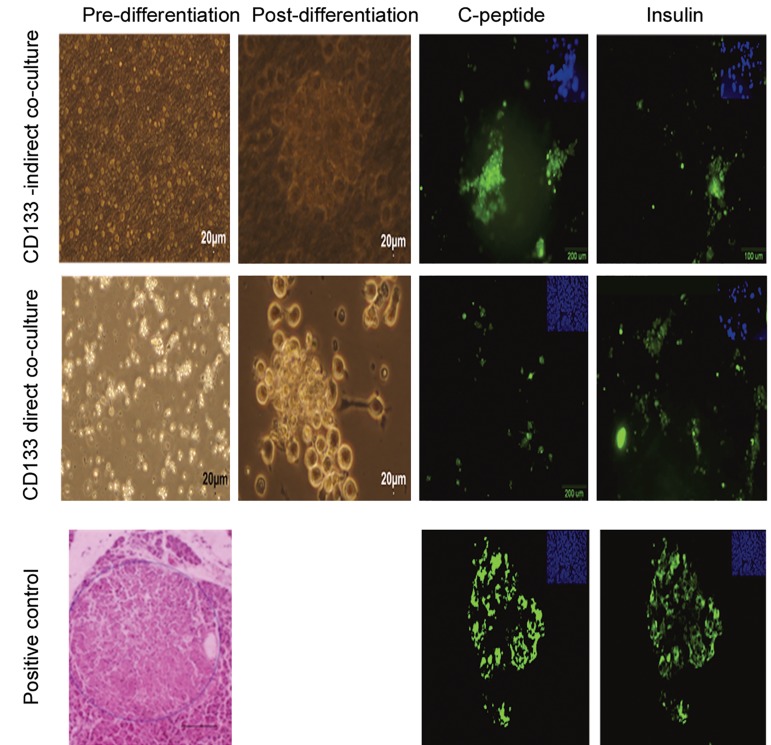
Effect of rat pancreatic mesenchymal cells on differentiation of umbilical cord blood cluster of differentiation (UCB-CD133^+^) into pancreatic β cells. Morphology and immunophenotyping of cells pre- and post-differentiation (×100). Immunofluorescence staining of cells for insulin (FITC) and C-peptide (FITC) in the groups co-cultured with rat pancreatic stromal cells. As observed with fluorescent microscope, insulin and C-peptide expressed after pancreatic differentiation in islet-like clusters. Human cadaver pancreas was the positive control. Nuclei were counterstained with 4΄,6-diamidino-2-phenylindole (DAPI).

**Fig.5:**
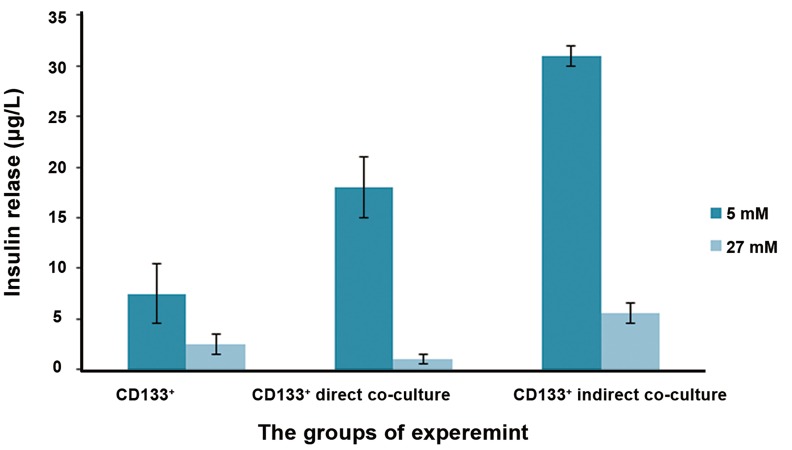
Functional assay on differentiated cells. Insulin release in response to low (5 mM) and high (27 mM) glucose concentrations in the final step of differentiation measured after a one hour incubation period. The difference between insulin secretion in response to low and high glucose concentrations in the tested groups was not statistically significant. All data are presented as means ± standard error (SE) from three independent experiments (P<0.05). The wavelength was 490 nm.

As shown in figure 4, the number of colonies was higher when UCB-CD133^+^ cells had cell-to-cell contact with rat PMCs compared to the indirect culture. The cells in both groups were positive for C-peptide and insulin when stained with monoclonal antibodies against these proteins. No significant difference could be observed in the expression of pancreatic specific genes *INSULIN, GLUCAGON, NKX6.1,* and *PDX1* in the presence or absence of rat PMCs at ten days post-induction. The differentiated cells from UCB-CD133^+^ cells did not secret valuable levels of insulin and C-peptide in the supernatant tested by ELISA (Fig.5) and in response to different concentrations of glucose. Unexpectedly, the highest levels of insulin secretion were observed in response to the supernatants that contained 5 μM of glucose (P<0.05). The undifferentiated cells (negative control) showed no significant release of insulin into the medium (data not shown).

## Discussion

The possibility of generating IPCs from various cellular sources including embryonic stem cells (11-15) and MSCs (16-21) has been recently demonstrated. However, the efficiency of differentiation and the normal function of these newly formed β-cells, especially in response to glucose stimulation are challengeable questions. Stromal niches can be thought of as discrete anatomical sites that contain niche support cells which physically contact adjacent cells and influence stem cell behavior via close range signaling (6). In this study we have differentiated UCB-CD133^+^ cells into IPCs both with and without rat PMCs in an attempt to investigate the effect of the pancreatic stromal niche on generating IPCs.

In the present study, we generated IPCs through a three step protocol and confirmed the presence of insulin production by immunofluorescence. The functionality of the *in vitro* generated IPCs was tested by measuring insulin and C-peptide release in response to glucose challenges.

Our results demonstrated that purified CD133^+^ cells expressed *CD133, OCT4* and *NANOG* genes which confirmed their stem potential. They easily formed islet-like colonies at the end of the differentiation process. The colonies expressed insulin and C-peptide at the protein level but were not functional and could not increase insulin production and release in response to high glucose concentrations.

We investigated the effect of the pancreatic stromal niche on generating IPCs from UCB-CD133^+^ cells by using a co-cultured system. Our finding showed no significant differences between cells cultured in the presence or absence of rat PMCs, however in direct culture the number of colonies increased. Other studies have also confirmed that rat pancreas extract could stimulate phenotypic pancreatic differentiation (8, 9). As our differentiated cells did not significantly express specific protein markers (insulin and C-peptide) and were unresponsive to high glucose concentrations, thus we proposed that pancreatic stromal cells might not be sufficient to induce functionally mature pancreatic differentiated cells as previously reported by Lumelsky et al. (22).

Apoptotic cells can take up exogenous insulin from culture medium. We have measured insulin and C-peptide protein to demonstrate de novo synthesis of insulin (12). We did not detect C-peptide in the pancreatic differentiation medium before differentiation (day 0, data not shown). Therefore, detectable levels of C-peptide in culture supernatants collected after differentiation should come from differentiated cells. Both differentiated cells (with and without co-culture) secreted insulin and C-peptide in response to glucose stimulations. However, there was no significant difference in secretion of insulin and C-peptide between these two groups. Glucose stimulation assays showed that IPCs generated by these groups released insulin and C-peptide in response to minimal glucose (5 μM) stimulation, yet did not release significant amounts of insulin and C-peptide at a higher glucose (27.5 μM) stimulation. IPCs might therefore merely release all of the insulin and C-peptide after stimulation with a low dose of glucose and have no insulin reserve to release upon re-stimulation at a higher dose of glucose. The time taken by IPCs to synthesize pro-insulin and process insulin might exceed the one hour time span in which the cells were incubated with the higher dose of glucose. Thus, IPCs could have released all of their intracellular insulin and had insufficient time to resynthesize more insulin for release during the incubation period at the higher glucose level. On the other hand, IPCs might not have been mature enough; therefore, the number of glucose receptors was not sufficient, which caused them to lack normal function in response to glucose stimulations. Additional, more extensive studies would be necessary to clarify the reasons for these observations.

## Conclusion

The data presented in this study indicated that human UCB-CD133^+^ cells could differentiate into IPCs *in vitro*. Pancreatic stromal cells might cause an enhancement in the number of immature pancreatic β-cells, but not in the number of mature cells. More research would be necessary to determine the role of other factors of the pancreatic niche on the differentiation of β-cells in generating IPCs *in vitro*.
