# *In Vitro* Differentiation of Human Umbilical Cord Blood CD133+ Cells into Insulin Producing Cells in Co-Culture with Rat Pancreatic Mesenchymal Stem Cells

**DOI:** 10.22074/cellj.2021.7935

**Published:** 2021-03-01

**Authors:** Fazel Sahraneshin Samani, Marzieh Ebrahimi, Tahereh Zandieh, Reyhaneh Khoshchehreh, Mohamadreza Baghaban Eslaminejad, Nasser Aghdami, Hossein Baharvand

**Affiliations:** 1. Department of Stem Cells and Developmental Biology at Cell Science Research Center, Royan Institute for Stem Cell Biology and Technology, ACECR, Tehran, Iran; 2. Department of Developmental Biology, University of Science and Culture, ACECR, Tehran, Iran; 3. Department of Regenerative Biomedicine at Cell Science Research Center, Royan Institute for Stem Cell Biology and Technology, ACECR, Tehran, Iran

In this article which was published in Cell J, Vol 17, No 2, Summer 2015, on pages 211-220, the authors found that Figures 3 and 4 had some errors that accidentally happened during organizing figures as well as because of mislabeling of some images and saving them in an incorrect folder. The following figures are corrected.

The authors would like to apologies for any inconvenience caused.

**Fig.3:**
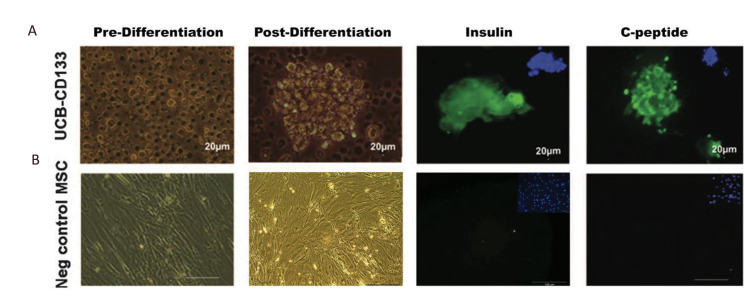
Immunofluorescence staining for insulin (FITC) and C-peptide (FITC) in differentiation umbilical cord blood cluster of differentiation 133+ (UCBCD133+) cells. **A. **The bright field images of pre- and post-differentiation UCB-CD133+ cells. Expressions of insulin and C-peptide conjugate with FITC (green) and nucleus stained with DAPI and **B.** Bright field images of mesenchymal stem cells (MSCs) pre- and postdifferentiation and lack of expressions of insulin and C-peptide in the cells (×100).

**Fig.4:**
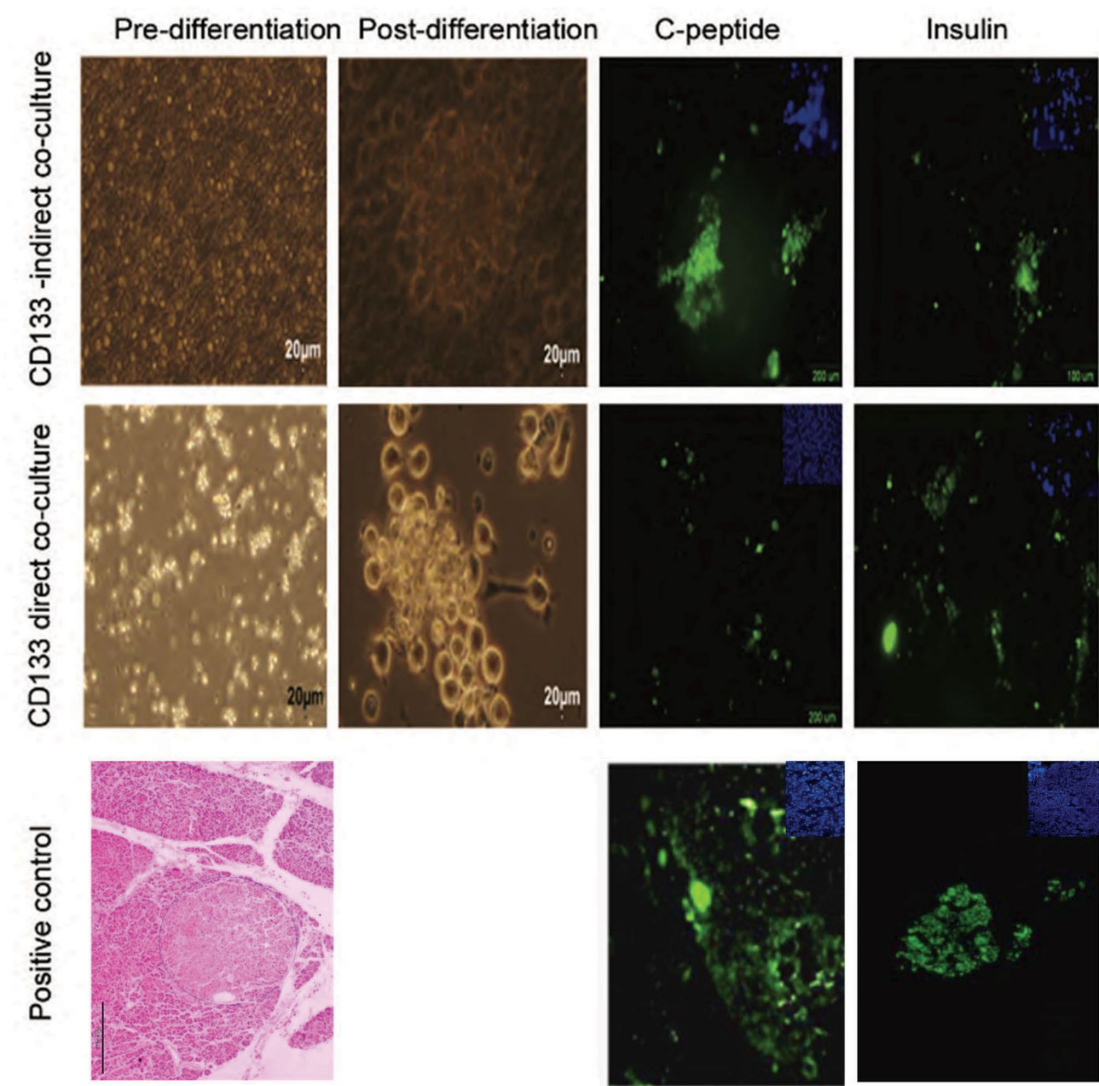
Effect of rat pancreatic mesenchymal cells on differentiation of umbilical cord blood cluster of differentiation (UCB-CD133+) into pancreatic β cells. Morphology and immunophenotyping of cells pre- and post-differentiation (×100). Immunofluorescence staining of cells for insulin (FITC) and C-peptide (FITC) in the groups co-cultured with rat pancreatic stromal cells. As observed with fluorescent microscope,insulin and C-peptide expressed after pancreatic differentiation in islet-like clusters. Human cadaver pancreas was the positive control. Nuclei were counterstained with 4΄,6-diamidino-2- phenylindole (DAPI).

